# C-terminal truncated hepatitis B virus X protein regulates tumorigenicity, self-renewal and drug resistance via STAT3/Nanog signaling pathway

**DOI:** 10.18632/oncotarget.15183

**Published:** 2017-02-08

**Authors:** Rachel Hiu Ha Ching, Karen Man Fong Sze, Eunice Yuen Ting Lau, Yung-Tuen Chiu, Joyce Man Fong Lee, Irene Oi Lin Ng, Terence Kin Wah Lee

**Affiliations:** ^1^ State Key Laboratory for Liver Research, The University of Hong Kong, Hong Kong; ^2^ Department of Pathology, Li Ka Shing Faculty of Medicine, The University of Hong Kong, Hong Kong; ^3^ Current address: Department of Applied Biology and Chemical Technology, Lee Shau Kee Building, Hong Kong Polytechnic University, Hong Kong

**Keywords:** truncated HBx, Nanog, Stat3, stemness, hepatocellular carcinoma (HCC)

## Abstract

Hepatitis B virus (HBV) is a major risk factor of chronic liver disease and hepatocellular carcinoma (HCC). Random integration of HBV DNA into the host genome is frequent in HCC leading to truncation of the HBV DNA, particularly at the C-terminal end of the HBV X protein (HBx). C-terminally truncated HBx (HBx-ΔC) has been implicated in playing a pro-oncogenic role in hepatocarcinogenesis. However, the mechanism whereby HBx-ΔC1 contributes to hepatocarcinogenesis remains unclear. In this study, we investigated the functional role of HBx-ΔC1 in regulating liver cancer stem cell (CSC) properties. Using Tet-on inducible system, we found that HBx-ΔC1 enhanced CSC properties including self-renewal, tumorigenicity, chemoresistance, migration and expression of liver CSC markers, when compared with the full-length HBx counterpart and vector control. Interestingly, HBx-ΔC1 conferred resistance in HCC cells towards sorafenib treatment through suppression of apoptotic cascade. In addition, HBx-ΔC1 upregulated a panel of stemness genes, in which Nanog was found to be among the most significant one in both trasnfected cell lines. Consistently, Nanog was upregulated in human HCC samples which had HBx-ΔC1 expression. Furthermore, the induction of CSC properties by HBx-ΔC1 was via the Stat3/Nanog pathway, as administration of Stat3 inhibitor abolished the HBx-ΔC1-induced self-renewing capacity. In conclusion, our data suggest that HBx-ΔC1 enhances liver CSCs properties through Stat3/Nanog cascade, and provide a new insight for the therapeutic intervention for HBV-related HCC.

## INTRODUCTION

Hepatocellular carcinoma (HCC) is the fifth most prevalent cancer and a highly lethal cancer with increasing worldwide incidence [1]. Chronic infection with hepatitis B virus (HBV) is one of the dominant etiological factors of HCC in Asia, accounting to 76% of all HCC cases [2]. HBV X protein (HBx), encoded by HBV X-gene, has been implicated to play a multifunctional oncogenic role in the development of HBV-associated HCC, such as promoting cell cycle progression, increasing cellular migration, regulating apoptotic process, inactivating negative growth regulators and inhibiting nucleotide excision repair of damaged cellular DNA [3, 4]. Accumulating evidence shows that random integration of HBV is detected in 80% to 90% of the host genome from HBV-infected HCC cases, frequently leading to truncation of HBV genome, especially on the HBx gene locus at the C-terminus [5, 6]. Our previous findings, together with other recent studies, have demonstrated that C-terminally truncated HBx (a major C-terminal truncated form reported with a breakpoint at 130aa) (HBx-ΔC1) may contribute to more aggressive behavior of HCC, which includes enhancing metastasis and tumorigenicity [7–10]. However, the molecular mechanism underlying HBx-ΔC1 contributes to HCC remains largely unknown. This lack of knowledge presents a hurdle to the identification of novel targeted therapeutic strategies for this fatal disease.

Recently, increasing evidence has emerged in support of the existence of cancer stem cell (CSC) / tumor-initiating cell (T-IC) model in leukemia and a wide range of solid tumors, including HCC [11]. CSCs are believed to possess both cancer cell- and stem cell-like characteristics, such as tumor initiation, self-renewal, differentiation and chemoresistance [12]. These cells are now regarded as the root of the tumor origin and recurrence. Specifically, in HCC, integrative comparative genomic analysis has established molecular similarities between CSCs and normal tissue stem cells, suggesting a role for liver CSCs in hepatocarcinogenesis [13]. Previously, we and others have demonstrated that CD24^+^ HCC cells functionally contributed to tumorigenicity and self-renewal though STAT3/Nanog pathway [14, 15]. Recently, HBx has been implicated to induce stem cell-like and CSC-like signatures in HCC [16, 17]. In addition, HBx has also been shown to enhance the expansion and tumorigenicity of hepatic progenitor cells (HPCs) in HBx transgenic mice [17], suggesting the importance of HBx in regulating cancer stemenss. Since HBx truncation is frequently observed in HBV-associated HCC, it is crucial to examine the role of truncated HBx in regulating liver CSCs in HCC.

In the present study, we revealed the functional role of HBx-ΔC1 in regulating liver CSCs. Our data showed that HBx-ΔC1 induced CSC properties including self-renewal, tumorigenicity, and drug resistance through the Stat3/Nanog cascade. Taken together, the current findings provide a better understanding on the molecular mechanism of HCC development associated with chronic HBV infection, and will significantly improve our knowledge of HCC pathogenesis with the goal of developing a more effective management for this deadly disease.

## RESULTS

### HBx-ΔC1 expressing cells increased the expression of stemness-related genes

**Figure 1:**
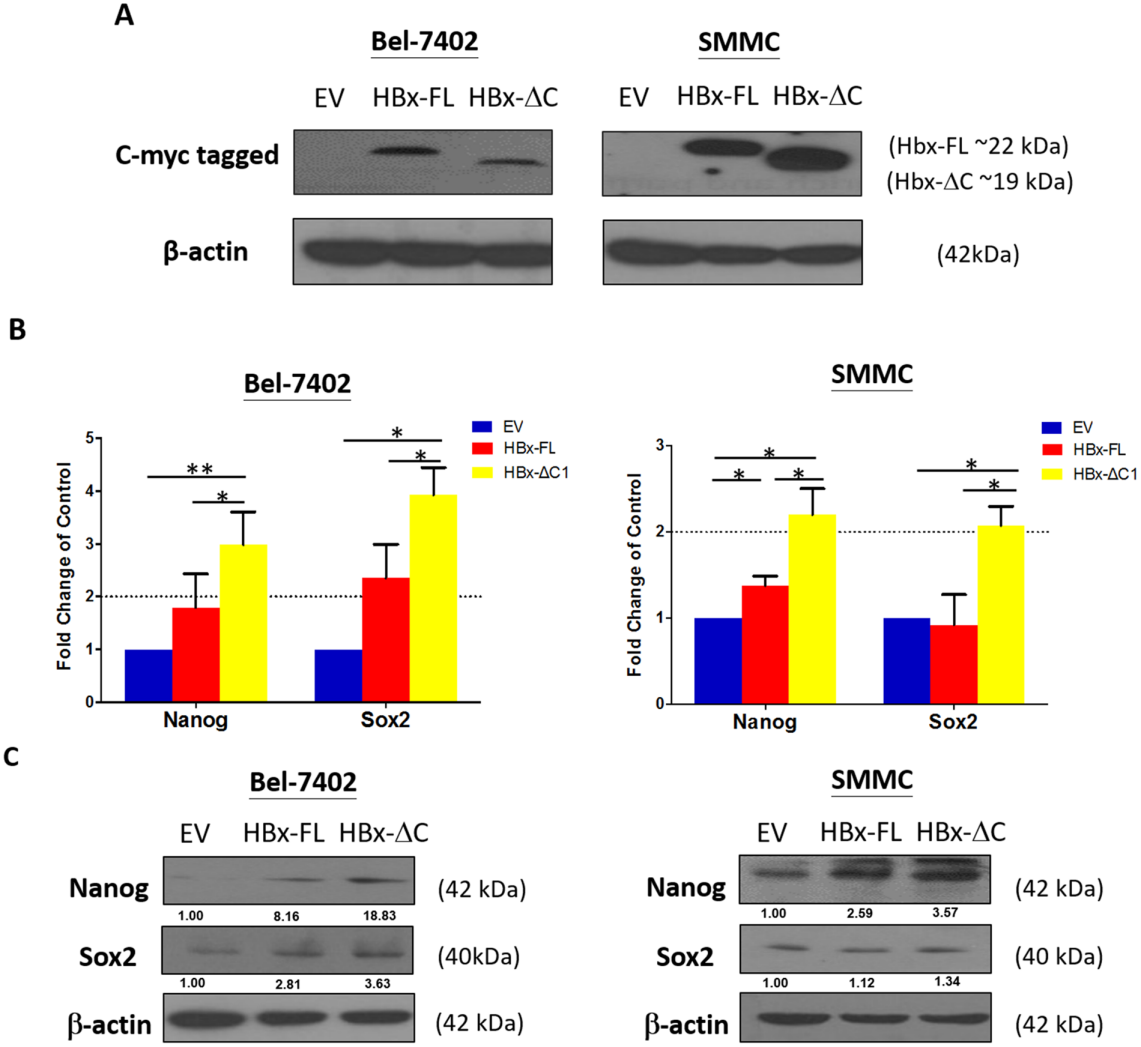
HBx-ΔC1 expressing cells increased expression of stemness-related genes **A**. Direct protein lysates of control and HBx expressing Bel-7402 and SMMC-7721 cells were collected after adding doxycycline for 23 and 6 days respectively and confirmed that HBx-FL and HBx-ΔC1 proteins were successfully expressed in respective transfected Bel-7402 and SMMC cells, by indirect immunoblotting using c-myc antibodies to detect myc-tagged-Hbx-FL and HBx-ΔC1 protein. **B**. qPCR analysis revealed HBx-ΔC1 expressing Bel-7402 had increased expression of stemness-related genes including of Nanog and Sox2 after adding doxycyclines for 23 days when compared with control (EV) and HBx-FL expressing Bel-7402. After adding doxycycline for 6 days, HBx-ΔC1 expressing SMMC-7721 cells had increased mRNA expression of Nanog and Sox2 when compared with EV and HBx-FL expressing SMMC-7721. **C**. Western blot analysis showed that Nanog was found to be preferentially expressed in transfectants of HBx-ΔC1, when compared with those with EV and HBx-FL.

Using lentiviral based Tet-On overexpression approach, we have successfully established inducible expression of HBx-FL and HBx-ΔC1 [8] in Bel-7402 and SMMC-7721 cells upon the addition of doxycycline at 1 μg/ml (Figure 1A). To determine the optimal time of gene induction, we examined the effect of HBx-FL and HBx-ΔC1 on the induction of stemness-related genes ranging from Day 5 to Day 27 and Day 6 to Day 9 in Bel-7402 and SMMC-7721 transfectants, respectively (Supplementary Figure 1). We found a stepwise increase in expression of stemness-related genes including Nanog and Sox2 from Empty vector (EV), HBx-FL to HBx-ΔC1 transfectants derived from Bel-7402 from Day 23 onwards (Figure 1B and Supplementary Figure 1). Similar effect on expression of stemness-related genes was observed in HBx-ΔC1 expressing SMMC-7721 transfectants with shorter doxycycline incubation time from day 6 onwards. (Figure 1B). To further verify this finding, we examined the expression of Nanog and Sox2 in transfectants of EV, HBx-FL, and HBx-ΔC1 in Bel-7402 and SMMC-7721 by western blot analysis (Figure 1C). However, only Nanog was found to be markedly increased in HBx-ΔC1 expressing Bel-7402 and SMMC-7721, when compared with EV and HBX-FL. Based on these data, it suggests the possible link between HBx-ΔC1 and Nanog.

### HBx-ΔC1 expressing cells were endowed with enhanced tumorigenicity, self-renewal, and expression of CSC markers

**Figure 2:**
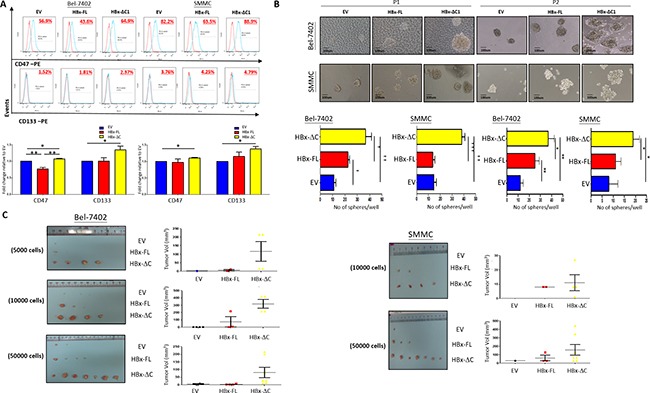
HBx-ΔC1 expressing cells possessed enhanced CSC properties **A**. Flow cytometry analysis revealed increased expression of CSC markers including CD47 and CD133 in HBx-ΔC1 clones when compared with control (EV) and HBx-FL clones in both Bel-7402 and SMMC-7721 (*p<0.05, **p<0.01, Student's t test). **B**. Spheroid formation after 8-10 days (10X objective) showed that both HBx-ΔC1 expressing Bel-7402 and SMMC-7721 cells had increased number and size of hepatospheres formed when compared with respective control (EV) and HBx-FL expressing cells (*p<0.05, **p<0.01, ***p<0.001, Student's t test). P1 and P2 = passage 1 and 2, respectively **C**. Control, HBx-FL- or HBx-ΔC1 expressing Bel-7402 and SMMC-7721 cells were injected subcutaneously at 4 sites (2 in the right flank and 2 in the left flank) per NOD/SCID mouse in three different cell numbers (5000, 10000, 50000 cells). HBx-ΔC1 expressing cells exhibited increased tumor-forming incidence and size when compared with EV and HBx-FL expressing cells. For SMMC-7721, no tumor was formed in all three clones when 5000 cells were injected (data not shown).

CSCs are believed to possess the stem/progenitor properties of expression of CSC markers [18], self-renewal [19], and tumorigenicity in immunodeficient mice [20]. Based on the results observed in Figure 1B and 1C, all functional CSC assays were performed based from Day 23 and Day 6 onwards upon doxycycline administration in Bel-7402 and SMMC-7721 cells, respectively. First, we found that overexpression of HBx-ΔC1 increased the expression of liver CSC markers including CD133 and CD47 when compared with control and HBx-FL expression in Bel-7402 and SMMC-7721 (Figure 2A). Accompanied with these changes, we found that HBx-ΔC1 transfectants exhibited increased self-renewal ability, as evidenced by the increase in the number and size of the spheres and their passages in sphere formation assay (Figure 2B). Next, we examined whether C-terminal truncated form of HBx was more tumorigenic than full-length HBx *in vivo* by tumor forming assay with HBx-FL expressing and HBx-ΔC1 expressing Bel-7402 and SMMC-7221 cells. Cells at density of 5000, 10000 and 50000 were inoculated subcutaneously into NOD/SCID mice. An increase in tumor incidence and size was observed in HBx-ΔC1 expressing cells when compared with control and HBx-FL expressing cells (Figure 2C).

### HBx-ΔC1 conferred chemoresistance and migratory properties to HCC cells

**Figure 3:**
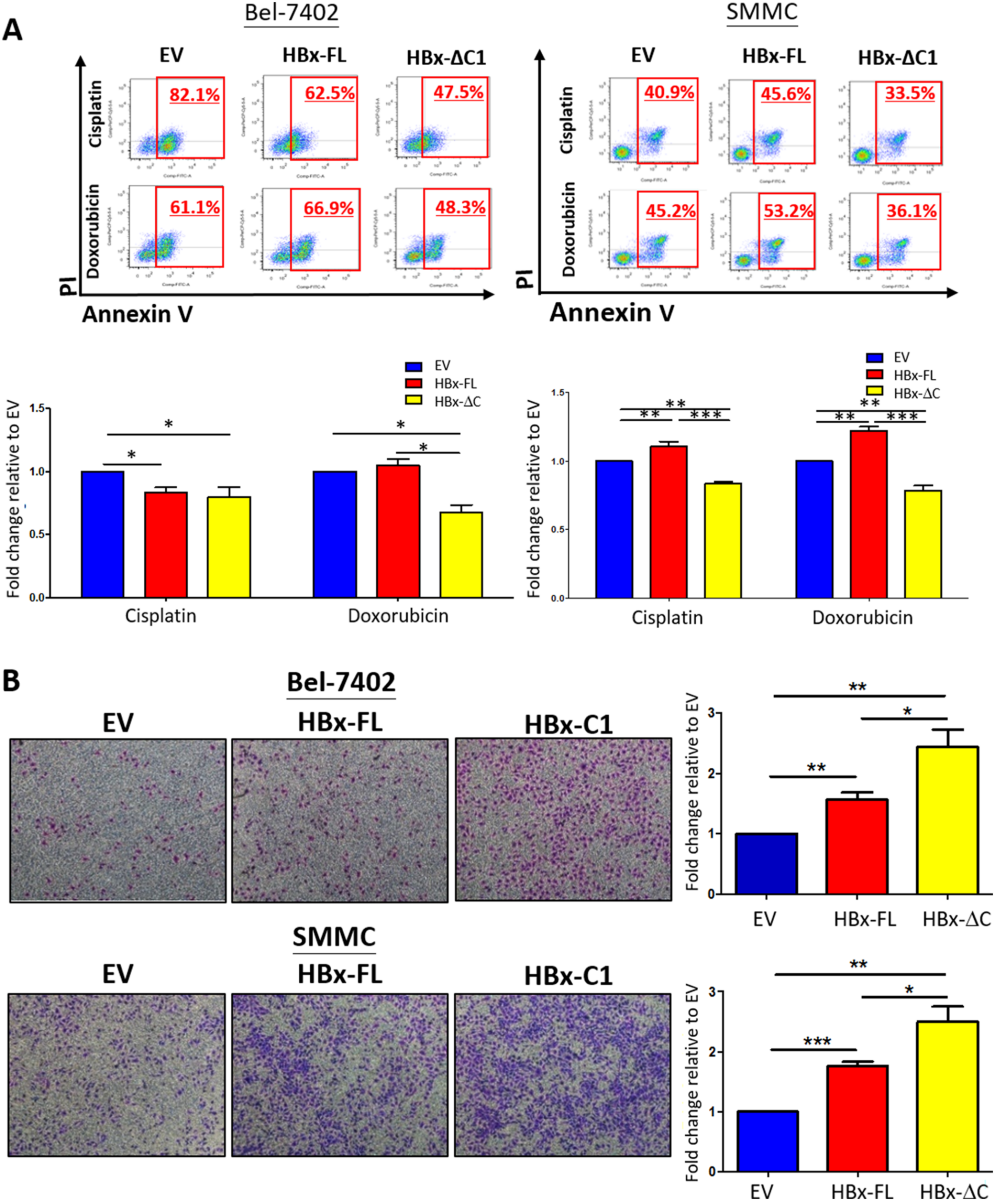
HBx-ΔC1 conferred resistance to chemotherapeutic drugs and enhanced cell migration **A**. HBx-ΔC1 overexpression conferred resistance to chemotherapeutic drugs including cisplatin and doxorubicin in both Bel-7402 and SMMC-7721, when compared to respective EV and HBx-FL, (Cisplatin: Bel-7402: 47.5% vs 82.1% and 62.5%; SMMC-7721: 33.5% vs 40.9% and 45.6%); and (Doxorubicin: Bel-7402: 48.3% vs 61.1% and 66.9%; SMMC-7721: 36.1% vs 45.2% and 53.2%) after treatment for 48hr at 0.25 and 2 μg/ml at 2% serum containing medium, 48hr at 4 and 10 μg/ml at 2% serum containing medium, respectively (*p<0.05, **p<0.01, ***p<0.001, Student's t test). **B**. Light micrograph of the cell migration assays, with staining of the cells in the lower chamber (4× objective), demonstrated that HBx-ΔC1 overexpression was more potent to enhance metastatic ability of cells in both Bel-7402 and SMMC-7721 clones (*p<0.05, **p<0.01, ***p<0.001, Student's t test).

Our previous studies have demonstrated that liver CSCs are more chemoresistant to chemotherapeutic drugs [21, 22]. Thus, we assessed whether HBx-ΔC1 conferred chemoresistance to HCC cells. For this purpose, we treated the transfectants of EV, HBx-FL and HBx-ΔC1 derived from Bel-7402 and SMMC-7721 with cisplatin and doxorubicin and subjected them to Annexin V staining assay. HBx-ΔC1 transfectants was found to be more chemoresistant to cisplatin and doxorubicin when compared with EV and HBx-FL transfectants in both Bel-7402 and SMMC-7721 cells (Figure 3A). In our previous study, we found that HBx-ΔC1 was significantly correlated with more aggressive phenotype of HCC patients [8]. Consistent with that clinical observation, we found that overexpression of HBx-ΔC1 conferred greater migratory ability to HCC cells, when compared with control and HBx-FL overexpression (Figure 3B).

### HBx-ΔC1 preferentially conferred resistance to sorafenib through suppression of apoptotic pathway

**Figure 4:**
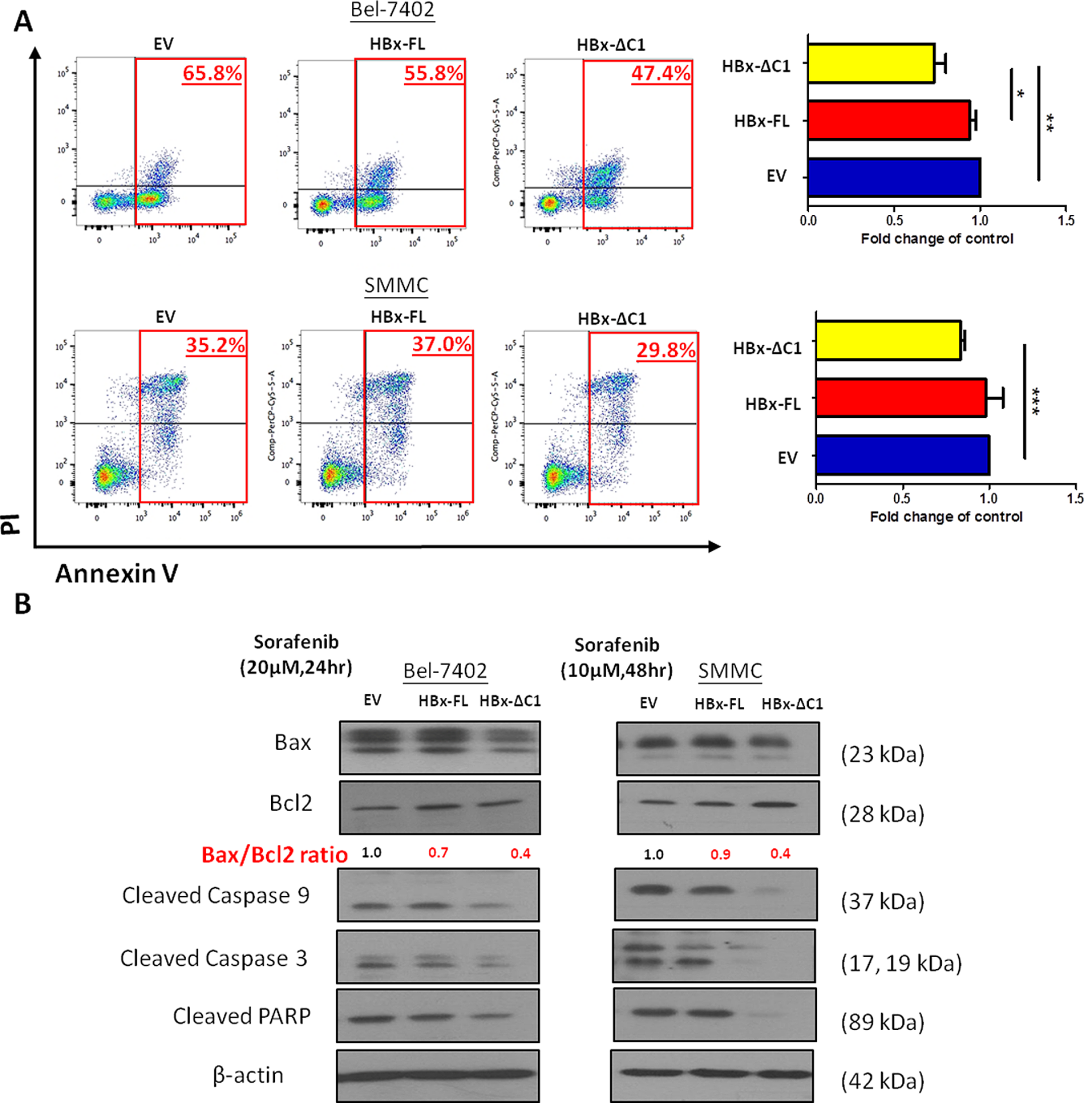
HBx-ΔC1 enhanced the resistance to sorafenib-induced apoptosis **A**. HBx-ΔC1 overexpression enhanced sorafenib resistance in both Bel-7402 and SMMC-7721, when compared to EV and HBx-FL expressing cells (Bel-7402: 47.4% vs 65.8% and 55.8%; SMMC-7721: 29.8% vs 35.2% and 37.0%), after treatment for 48hr at 10 and 20 μM at 5% serum containing medium respectively (*p<0.05, **p<0.01, ***p<0.001, Student's t test). **B**. HBx-ΔC1 overexpression reduced apoptotic signaling pathway by decreasing the Bax/Bcl2 ratio, indicator of apoptosis, the expression of activated cleaved form of caspase 9 and 3 and PARP when compared to control and HBx-FL expressing cells.

Previously, we found that liver CSCs were enriched upon sorafenib treatment, as evidenced by an increase in the abilities in self-renewal and tumorigenicity in sorafenib-resistant cells [22]. Since HBx-ΔC1 upregulated the traits of liver CSCs as shown in Figures 2 and 3, we hypothesized that HBx-ΔC1 conferred resistance to sorafenib treatment. For this purpose, we compared the sensitivity towards sorafenib among HBx-FL and HBx-ΔC1 transfectants of Bel-7402 and SMMC-7721 and their controls. By Annexin V staining, HBx-ΔC1 transfectants was found to be more resistant to sorafenib when compared with EV and HBx-FL transfectants in both Bel-7402 and SMMC-7721 cells (Figure 4A). To further understand the underlying molecular mechanism for sorafenib resistance conferred by HBx-ΔC1, we examined the expression of apoptosis cascade proteins including Bax, Bcl2, and cleaved forms of PARP, caspase-3 and 9 in the transfectants of EV, HBx-FL and HBx-ΔC1 derived from Bel-7402 and SMMC-7721 in response to sorafenib treatment. As shown in Figure 4B, HBx-ΔC1 transfectants demonstrated decreased Bax/Bcl2 ratio and expression of cleaved forms of PARP, capcase-3 and -9, when compared with EV and HBx-FL transfectants in both Bel-7402 and SMMC-7721 cells.

### HBx-ΔC1 driven tumor initiation and self-renewal through Stat3/Nanog signaling

**Figure 5:**
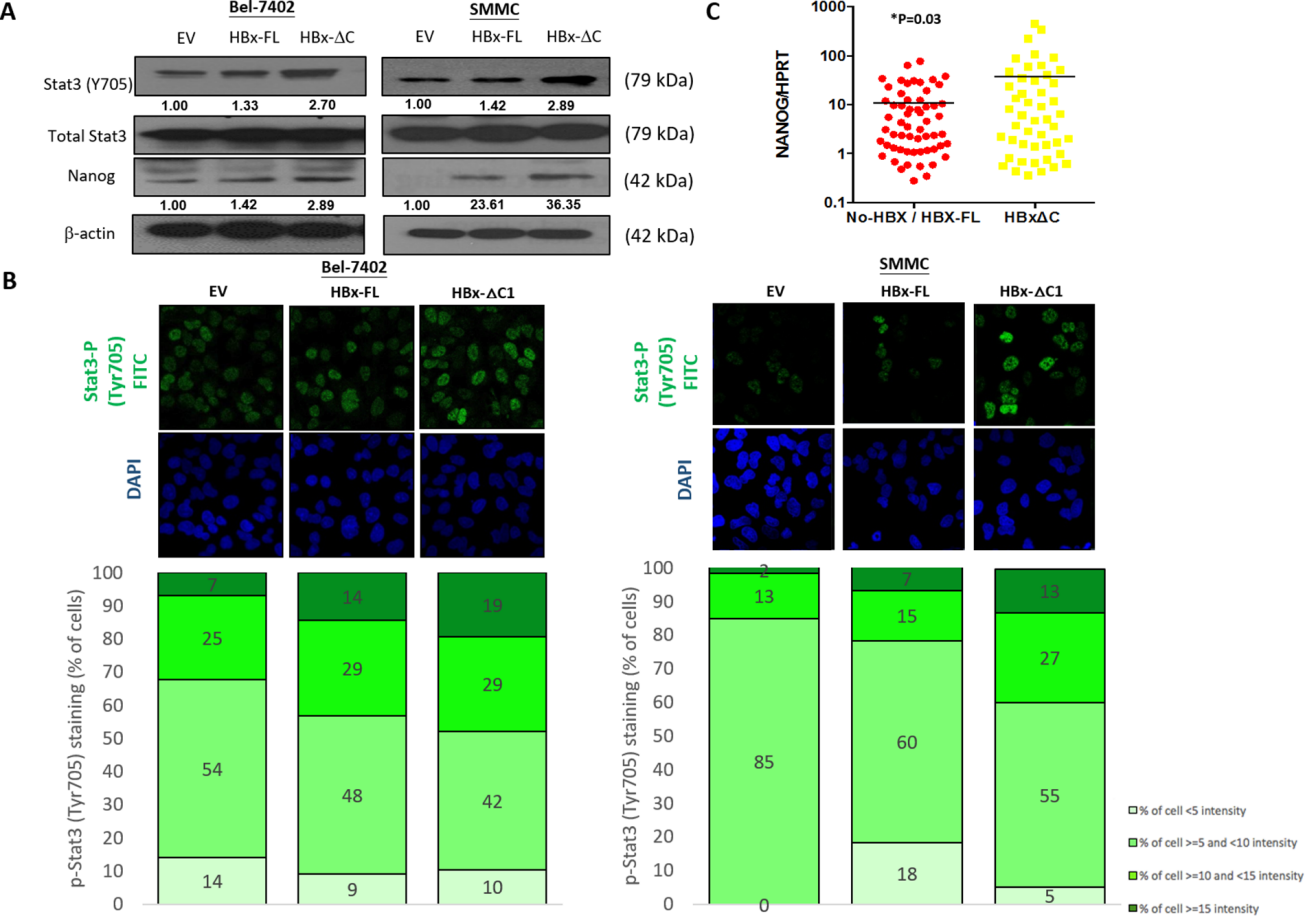
HBx-ΔC1 regulated liver CSC properties through Stat3-Nanog signaling **A**. Western blot analysis revealed that HBx-ΔC1 expressing Bel-7402 and SMMC-7721 cells had increased Stat3 phosphorylation (Y705) and expression of Nanog, when compared to respective EV and HBx-FL expressing cells. **B**. Increased fluorescent nuclear staining of p-Stat3 (Y705) was observed in HBx-ΔC1 cells in both Bel-7402 and SMMC-7721when compared with respective EV and HBx-FL, as analyzed by immunefluorescence (IF) staining (40X objective). Stained cells were classified into 4 groups as per the fluorescent intensity measured by Photoshop CS5. **C**. qPCR analysis demonstrated that the expression of Nanog was up-regulated in HCC samples detected with HBx-ΔC1 (n=48) when compared with those with HBx-negative and HBx-FL (n=59) (*p=0.03, t test).

**Figure 6:**
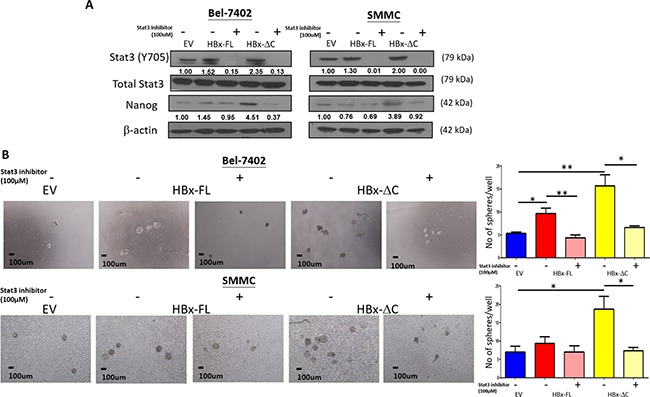
Effect of Stat3 inhibitor on HBx-induced self-renewal **A**. Nanog expression was found to be down-regulated upon treatment with S3I-201 for 24hr, as analyzed by western blot analysis. **B**. Pre-treatment of S3I-201 at the concentration of 100 and 150μM led to abolishment of HBx-ΔC1-induced self-renewal indicated by sphere forming assay where the number and size of sphere formed (4X objective) were significantly reduced in HBx-ΔC1 expressing Bel-7402 and SMMC-7721 cells (*p<0.05, **p<0.01, Student's t test).

Previously, we found that Stat3/Nanog pathway was activated in liver CSCs contributing to tumorigenicity and stemness [15]. In addition, Stat3 signaling activity has been shown to be enhanced in HBx expressing cells and HBx transgenic mice leading to carcinogenesis [17]. Therefore, we investigated if Stat3-mediated Nanog regulation was involved in the enhancement of cancer stemness by truncated HBx. By western blotting, we found that HBx-ΔC1 preferentially induced the expression of Stat3 (Y705) and stem cell transcriptional factor, Nanog, in Bel-7402 and SMMC-7721 (Figure 5A). We also determined the Stat3 activity by quantifying the phospho-Stat3 (p-Stat3 [Y705]) level using immunofluorescence staining. Consistently, HBx-ΔC1 stimulated Stat-3 activity as indicated by higher percentage of p-Stat3 (Y705) staining when compared with control and HBx-FL (Figure 5B). We also examined the expression of a panel of stemness genes in the HCC clinical samples by qPCR (Supplementary Figure 2). Consistently, Nanog expression was found to be up-regulated in the tumor samples with detectable expression of HBx-ΔC1 when compared to those with full-length HBx or without HBV infection (Figure 5C). To further examine whether the HBx-induced upregulation of Nanog was Stat3-dependent, we examined the p-Stat3 (Y705) and Nanog expression in the presence of a Stat3 inhibitor (S3I-201) in Bel-7402 and SMMC-7721 cells. By western blot analysis, Nanog expression was found to be downregulated upon S3I-201 treatment (Figure 6A). Furthermore, addition of S3I-201 abolished the HBx-induced self-renewal as indicated by sphere forming assay (Figure 6B). Taken together, these findings suggest that HBx-ΔC1 enhances liver CSCs properties through Stat3-mediated Nanog upregulation.

## DISCUSSION

HBV DNA is often integrated and highly rearranged within the host DNA in HCC [23]. Among the four proteins translated by HBV, the X-gene product (HBx), active in transactivation assay [24], has been closely associated with the HCC carcinogenesis. In the present study, we demonstrated that the expression of HBx-ΔC1 contributed to cancer stemness and tumorigenicity of HCC, providing novel evidence for the role of HBx-ΔC1 in HBV-infected liver cancer.

Recently, hepatitis B virus-infected hepatocytes induced a CSC-like gene signature with EpCAM upregulation [25]. In addition, HBx enhanced expression of stemness and CSC markers including Oct4, Nanog, Klf-4, and EpCAM *in vitro* and enhanced the expansion and tumorigenicity of hepatic progenitor cells (HPCs) in HBx transgenic mice [16, 17]. Consistent with the previous findings, we found that HBx enhanced the expression of stemness genes including Nanog and Sox2. Since COOH-truncated form of HBx has been shown to have more aggressive behavior in the development of HCC [7–10], in this study, in addition to full-length HBx, we also studied the role of COOH-truncated HBx, with a breakpoint at 130aa (HBx-ΔC1) [5, 6, 8, 26] in the regulation of cancer stemness. Interestingly, we demonstrated that HBx-ΔC1 enhanced the liver CSC properties including increased expression of stemness genes, self-renewal capacity, resistance to chemotherapeutic drugs and tumorigenesis, all these effects being more prominent than the full-length HBx. These results have demonstrated the distinct role of HBx-ΔC1 in promoting liver CSC properties.

Sorafenib is the only FDA-approved molecularly targeted drug shown to improve the survival of unresectable HCC. However, the survival benefit of the sorafenib treatment arm was on average only 2-3 months longer [27], partly due to drug resistance. In order to understand the molecular mechanism of sorafenib resistance, we previously established sorafenib-resistant HCC cells which were found to exhibit liver CSC properties including enhanced self-renewal capacity and tumorigenicity, when compared with their mock counterparts [22]. Since HBx-ΔC1 was found to upregulate liver CSC properties, we examined whether it conferred resistance to sorafenib treatment though enhancement of liver CSC properties. We found that HBx-ΔC1 suppressed the apoptotic cascade and thereby decreased the sensitivity of HCC cells towards sorafenib treatment. In this regard, HBx-ΔC1 may be a potential predictor for sorafenib response.

Stat3 activation has been found to be important in the maintenance of survival of liver CSCs (14). We and others have previously demonstrated that Nanog is the direct downstream effector of Stat3 in the regulation of tumorigenicity and self-renewal of liver CSCs and glioblastoma stem cells [15, 28]. Nanog is a self-renewal gene that maintains the pluripotency of embryonic stem cells [29]. Recently, it has been identified as a novel oncogene by a large-scale oncogenomics analysis [30], and it is also crucial in regulating human tumor development [31]. In addition, Stat3 signaling activity has been demonstrated to be enhanced in HBx expressing cells and HBx transgenic mice leading to carcinogenesis [17, 32]. Consistently, we found the upregulation of nuclear Stat3, p-Stat3 and Nanog expression in HBx-expression cells (Figure 4A and 4B). We found the Stat3/Nanog signaling pathway was preferentially activated in HBx-ΔC1 trasnfectants, which was indicated by the enhanced activity of Stat3 and expression of Nanog observed in HBx-ΔC1 expressing Bel-7402 and SMMC-7221 cells, when compared with HBx-FL and control cells. Furthermore, the role of HBx-ΔC1-induced self-renewal was confirmed by the treatment with Stat3 inhibitor. We found that the inhibition of Stat3 activation using a Stat3 specific inhibitor abrogated the effect of the HBx-ΔC1-induced self-renewal capacity. These findings suggest that Stat3-Nanog signaling plays a crucial role in regulating the stemness properties mediated by HBx-ΔC1.

In summary, the present findings demonstrated that HBx-ΔC1 plays a critical role in HCC development and progression via the regulation of cancer stemness, which involves the preferential activation of the Stat3-Nanog pathway. A better understanding of the molecular mechanism of HBx-ΔC1 will improve our knowledge of HCC pathogenesis with the goal of developing a more effective management for this deadly disease. Our data also give a new insight on developing targeted therapies against HBx-ΔC1-induced Nanog and the identification of novel markers to predict disease outcome and tumor recurrence.

## MATERIALS AND METHODS

### Plasmids

Full-length HBx DNA (GenBank no.: U95551) was amplified from the HBx/pcDNA3.1+ plasmid [8] and sub-cloned into Myc/pLVX-Tight Puro and Myc/pcDNA3.1+ vectors. HBx truncation mutant (named HBx-ΔC1) with 24 C-terminal amino acid of HBx deleted was made and sub-cloned into Myc/pLVX-Tight Puro and Myc/pcDNA3.1+ vectors.

### Cell lines

The human HCC cell lines Bel-7402 and SMMC-7721 (SIBS, Chinese Academy of Sciences) were grown in Dubecco's modified Eagle minimal high glucose essential medium (DMEM-HG) (Gibco-BRL) supplemented with 10% fetal bovine serum (FBS) (Gibco-BRL) and 1% penicillin and streptomycin (Gibco-BRL).

### Human HCC samples

107 human HCCs liver tissues from patients with liver resection for HCC between 1992 and 2010 at Queen Mary Hospital, Hong Kong, were randomly selected for study, in which, 102 patients had positive serum hepatitis B surface antigen (HBsAg) status. Patients' ages ranged from 27 to 74 years; 68 were male and 39 female. All specimens collected were either snap-frozen in liquid nitrogen and stored at -80°C, or fixed in 10% formalin for paraffin embedding. Use of human specimens was approved by the institutional review board of the University of Hong Kong/Hospital Authority Hong Kong West Cluster.

### Establishment of tetracycline-inducible HBx expressing cells

The Bel-7402 and SMMC-7721 cell line was, first, transfected with pLVX Tet-On Advanced vector (Clontech Laboratories, Inc., Mountain View, CA) using Lipofectamine 2000 (Invitrogen), according to manufacturer's protocol. tTA (Tet-On) expressing Bel-7402 and SMMC-7721 cells were selected with G418 at 700 μg/mL and 400 μg/mL for 14 days, respectively. To obtain stable inducible HBx expressing cells, lentivirus containing full-length and C-terminal truncated HBx in Myc/pLVX-Tight Puro vector was infected into tTA expressing Bel-7402 and SMMC-7721 cells and selected with puromycin at 1 μg/mL for 7 days.

### Statistical analysis

All statistical analyses were performed using the statistical software SPSS 17 for Windows (SPSS Inc., Chicago, IL). Student's *t* was used for continuous data wherever appropriate. *p* values less than 0.05 were considered significant.

Additional experimental procedures are provided in the Supporting Information.

## SUPPLEMENTARY MATERIALS AND METHODS FIGURES



## Abbreviations

HBV: Hepatitis B virus
HCC: Hepatocellular carcinoma
HBx: HBV X protein
HBx-ΔC1: C-terminally truncated HBx
CSC: cancer stem cell
