# Supplementary material for: C-terminal truncated hepatitis B virus X protein regulates tumorigenicity, self-renewal and drug resistance via STAT3/Nanog signaling pathway
Source: Oncotarget. 2017 Feb 8;8(14):23507–16. doi: 10.18632/oncotarget.15183 (PMC5410322; doi:10.18632/oncotarget.15183)
Supplement: Supplementary file 1 [file oncotarget-08-23507-s001.pdf]

C-terminal truncated hepatitis B virus X protein regulates tumorigenicity, self-renewal and drug resistance via STAT3/Nanog signaling pathway

SUPPLEMENTARY MATERIALS AND METHODS

Quantitative PCR (qPCR) analysis

Total RNA was isolated using Trizol reagent according to the manufacturer's protocol (Invitrogen, Carlsbad, CA). Complementary DNA (cDNA) was synthesized using a GeneAmp® Gold RNA PCR Kit (Applied Biosystems, Foster City, CA) according to the manufacturer's instructions and then subjected to PCR with a SYBR Green PCR kit with primers specific to the sequences of which are provided in Supplementary Methods. The amplification protocol consisted of incubations at 94°C for 15 seconds, 63°C for 30 seconds, and 72°C for 60 seconds. Incorporation of the SYBR Green dye into PCR products was monitored in real time with an ABI 7900HT Sequence Detection System and SDS 1.9.1 software (Applied Biosystems) and subsequently analyzed using RQ Manager 1.2 software (Applied Biosystems), thereby allowing the threshold cycle (C_T) at which exponential amplification of the products began to be determined. The amount of target cDNA was calculated relative to that of β-actin cDNA.

Primer Sequences

Genes	Forward primer(5′-3′)	Reverse primer(5′-3′)
GAPDH	CCGGGAAACTGTGGCGTGATGG	AGGTGGAGGAGTGGGTGTCGCTGTT
BMI1	TGGAGAAGGAATGGTCCACTTC	GTGAGGAAACTGTGGATGAGGA
Nanog	CCTGTGATTTGTGGGCCTG	GACAGTCTCCGTGTGAGGCAT
Nestin	GGCGCACCTCAAGATGTCC	CTTGGGGTCCTGAAAGCTG
Notch1	CCTGAGGGCTTCAAAGTGTC	CGGAACTTCTTGGTCTCCAG
Oct4	CTTGCTGCAGAAGTGGGTGGAGGAA	CTGCAGTGTGGGTTTCGGGCAC
Sox2	AAATGGGAGGGGTGCAAAAGAGGAG	CAGCTGTCATTTGCTGCTGTGGGTGATG
SMO	TGGTCACTCCCCTTTGTCCTCAC	GCACGGTATCGGTAGTTCTTGTAGC
b-catenin	ACAAACTGTTTTGAAAAATCCA	CGAGTCATTGCATACTGTCC
c-myc	CGTCCTCGGATTCTCTGCTC	GCTGGTGCATTTTCGGTTGT
KLF4	ACATGGCTGTCAGCGACGCG	TCCCGCCAGCGGTTATTCGGG
ABCG2	TCATCAGCCTCGATATTCCATCT	GGCCCGTGGAACATAAGTCTT
ABCB5	TCTGGCCCCTCAAACCTCACC	TTTCATACCGCCACTGCCAACTC
CD24	TGAAGAACATGTGAGAGGTTTGAC	GAAAACTGAATCTCCATTCCACAA
CD44	TGCCGCTTTGCAGGTGTAT	GGCCTCCGTCCGAGAGA
CD47	CAATCACGTAAGGGTCTCATAGG	GATGGACTCCGATTTGGAGA
CD133	TGGATGCAGAACTTGACAACGT	ATACCTGCTACGACAGTCGTGGT
EpCAM	CCATGTGCTGGTGTGTGAAC	ACGCGTTGTGATCTCCTTCT

Migration and invasion assays

Cell migration was investigated using transwell inserts with 6.5 mm polucarboate membranes of 8.0µm pore size (Corning Inc., NY, USA). Cells in serum-free medium were seeded onto the upper chamber of the transwell and the culture medium containing 10% FBS was added into the lower chamber serving as chemoattractant. Cells were incubated for 24hr to allow for migration through the membrane. Migrated cells were then fixed by 2% paraformaldehyde and stained with crystal violet. Stained cells were captured randomly for three fields under a light microscope and ells were counted. The experiments were repeated independently three times. The cell invasion assay was performed with self-coated Matrigel (BD Biosciences, San Jose, CA) on the upper surface of a transwell chamber. The invasive cells that had invaded through the extracellular matrix layer to the lower surface of the membrane were fixed with 2% paraformaldehyde and stained with crystal violet. Photographs of three randomly selected fields of the fixed cells were captured and cells were counted. The experiments were repeated independently three times.

Sphere formation assay

Cells were seeded into a 24-well plate pre-coated with 1% Poly (2-hydroxyethyl methacrylate) (Sigma-Aldrich, St. Louis, MO, USA) at 400-500 cells per well. Each well initially contained 350µl 0.25% Methyl cellulose (Sigma-Aldrich, St. Louis, MO, USA) in DMEM/F12 medium (Invitrogen) supplemented with B27 (Invitrogen), 20ηg/ml basic fibroblastic growth factor (bFGF), 20ηg/ml epidermal growth factor (EGF), 4µg/ml insulin (Sigma-Aldrich, St. Louis, MO, USA), 0.4% Bovine Serum Albumin (Roche, Germany), 1µg/ml doxycycline (BD Biosciences, San Jose, CA). 60µl of the supplemented medium was added per well every other day for approximately 8-10 days. The hepatospheres formed were captured under a light microscope. The number of hepatospheres were counted and the average number of hepatospheres per well was recorded.

RNA extraction and semi-quantitative reverse-transcriptase polymerase chain reaction

Total RNA of human HCC liver tissues was extracted using TRIzol (Invitrogen, Carlsbad, CA), according to manufacturer's protocol. For polymerase chain reaction (PCR) amplification of HBx, sets of PCR primers (1425F: 5′-TCCTTTGTTTACGTCCCGTC-3′, 1840R:-5′- TTAGGCAGAGGTGAAAAAGTTG-3′ and 1661R: 5′-GAATTCCTTATGTAAGACCTTGGGCAA CAT-3′) were used for full-length and COOH-truncated HBx, respectively.

In vivo tumorigenicity experiments

Tumorigenicity was evaluated *in vivo* by subcutaneous xenograft tumor models. Various number of HCC cells were subcutaneously injected at 4 sites (2 in the right flank and 2 in the left flank) into the non-obese diabetic (NOD)/ immune-deficient (SCID) mice to induce xenograft tumor formation. After injection, mice were administrated with 0.2mg/ml doxycycline (BD Bioscience) dissolved in drinking water containing 2% sucrose, and were under observation for tumor growth up 8 wk.

Flow cytometry analysis

Phycoerythrin (PE)-conjugated CD133, CD47 and CD24 cell surface markers or its isotype-matched mouse immunoglobulins (BD Biosciences, San Jose, CA; Miltenyl Biotec, Auburn, CA) were used to stain cells in freshly prepared phosphate-buffered saline (PBS) containing 2% FBS at 4°C for 30-45 min. Samples were then washed with PBS and analyzed with FACSCantoII flow cytometer and FACSDiva software (BD Biosciences, San Jose, CA) with a minimum acquisition of 10,000 events per sample.

Annexin V/propidium iodide apoptosis assay

Apoptosis, or programmed cell death, was determined by using Propidium iodide (PI) in conjunction with Annexin V. Cells were stained with FITC-conjugated Annexin V and PI at 5µl and 10µl per test, respectively, in 1x Annexin V binding buffer from FITC Annexin V Apoptosis Detection Kit (BD Biosciences, San Jose, CA) in dark at room temperature for 15 min. Apoptotic cells were detected and analyzed using FACSCantoII flow cytometer and FACSDiva software (BD Biosciences, San Jose, CA).

Western blot analysis

Western blots were developed using an ECL Plus kit (Amersham Biosciences, Piscataway, NJ). The primary antibodies included rabbit polyclonal anti-Nanog (Cell signaling technology, Danvers, MA, USA), rabbit polyclonal anti-Sox2 (Abcam, Cambridge, UK), rabbit monoclonal anti-Stat3 (Cell signaling technology, Danvers, MA, USA), rabbit monoclonal anti-Phospho-Stat3 (Tyr705) (Cell signaling technology, Danvers, MA, USA), rabbit polyclonal anti-Bcl2 (Cell signaling technology, Danvers, MA, USA), rabbit polyclonal anti-Bax (Santa Cruz Biotechnology, Dallas, Texas, USA), rabbit monoclonal anti-cleaved caspase-3 (Asp175) (Cell signaling technology, Danvers, MA, USA), rabbit monoclonal anti-cleaved caspase-9 (Asp330) (Cell signaling technology,

Danvers, MA, USA), rabbit monoclonal anti-cleaved PARP (Asp214) (Cell signaling technology, Danvers, MA, USA), mouse polyclonal anti-Actin (Sigma-Aldrich, St. Louis, MO, USA). After washing, the membrane was incubated with horseradish peroxidase-conjugated anti-mouse or rabbit antibody (Amersham) and then visualized with enhanced chemiluminescence plus according to the manufacturer's protocol.

Immunofluorescence microscopy

Cells were seeded onto coverslips and incubated overnight at 37°C in a CO2 incubator, then were fixed and permeablized with ice cold absolute methanol. Fixed cells were blocked with 3% BSA for 1 hour and then incubated with 1:50 rabbit monoclonal anti-Phospho-Stat3 (Tyr705) (Cell signaling technology, Danvers, MA, USA). Cells were counterstained with 4′,6-diamidino-2-phenylindole (DAPI) (Calbiochem, San Diego, CA) and mounted with Vectashield antifade mountant (Vector Laboratories, Burlingame, CA). Images were captured under 40× magnification by a fluorescence microscope (Carl Zeiss LSM 510 Meta/Axiocam).

SUPPLEMENTARY FIGURES

(A)

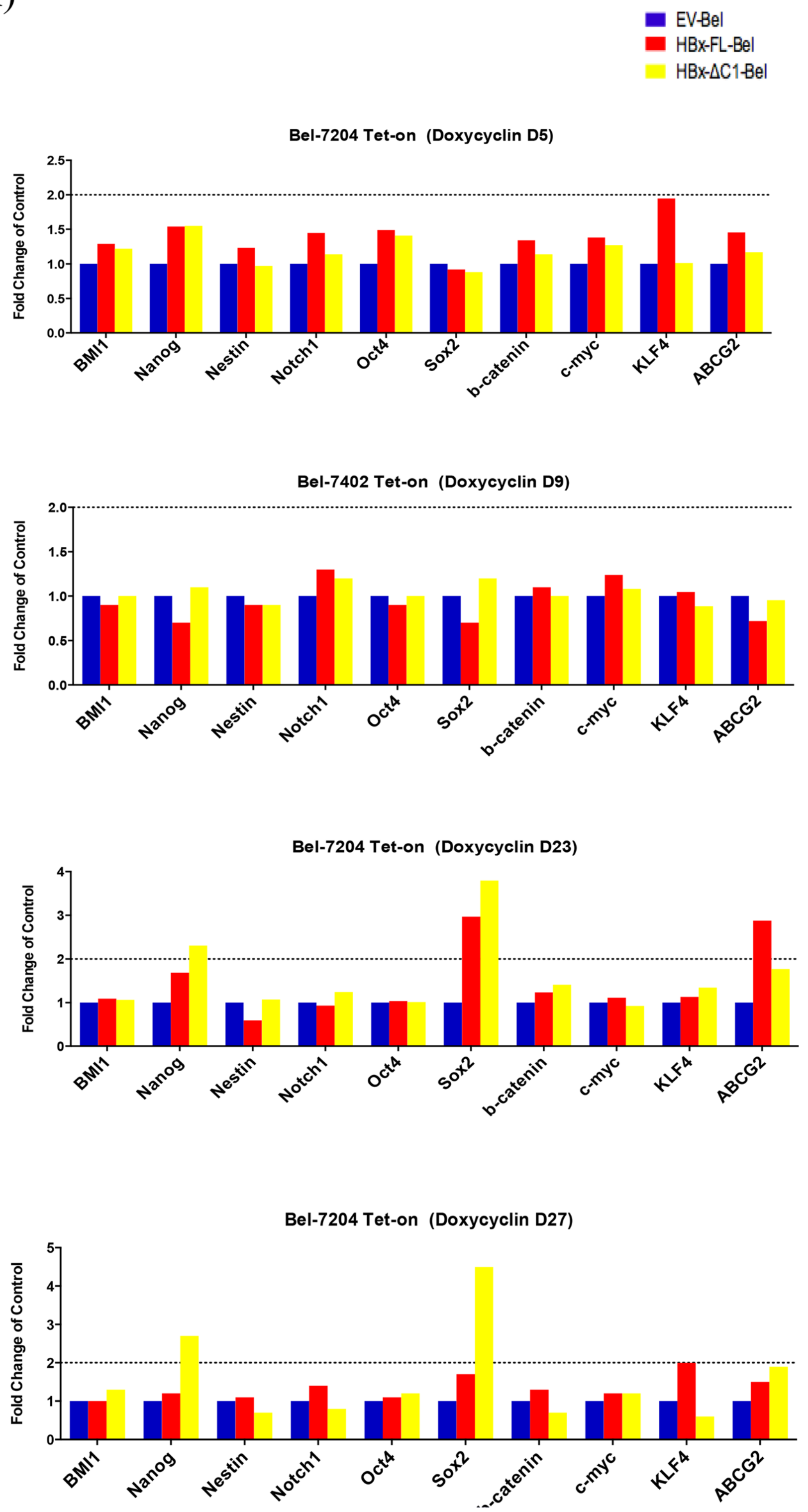

(*Continued*)

(B)

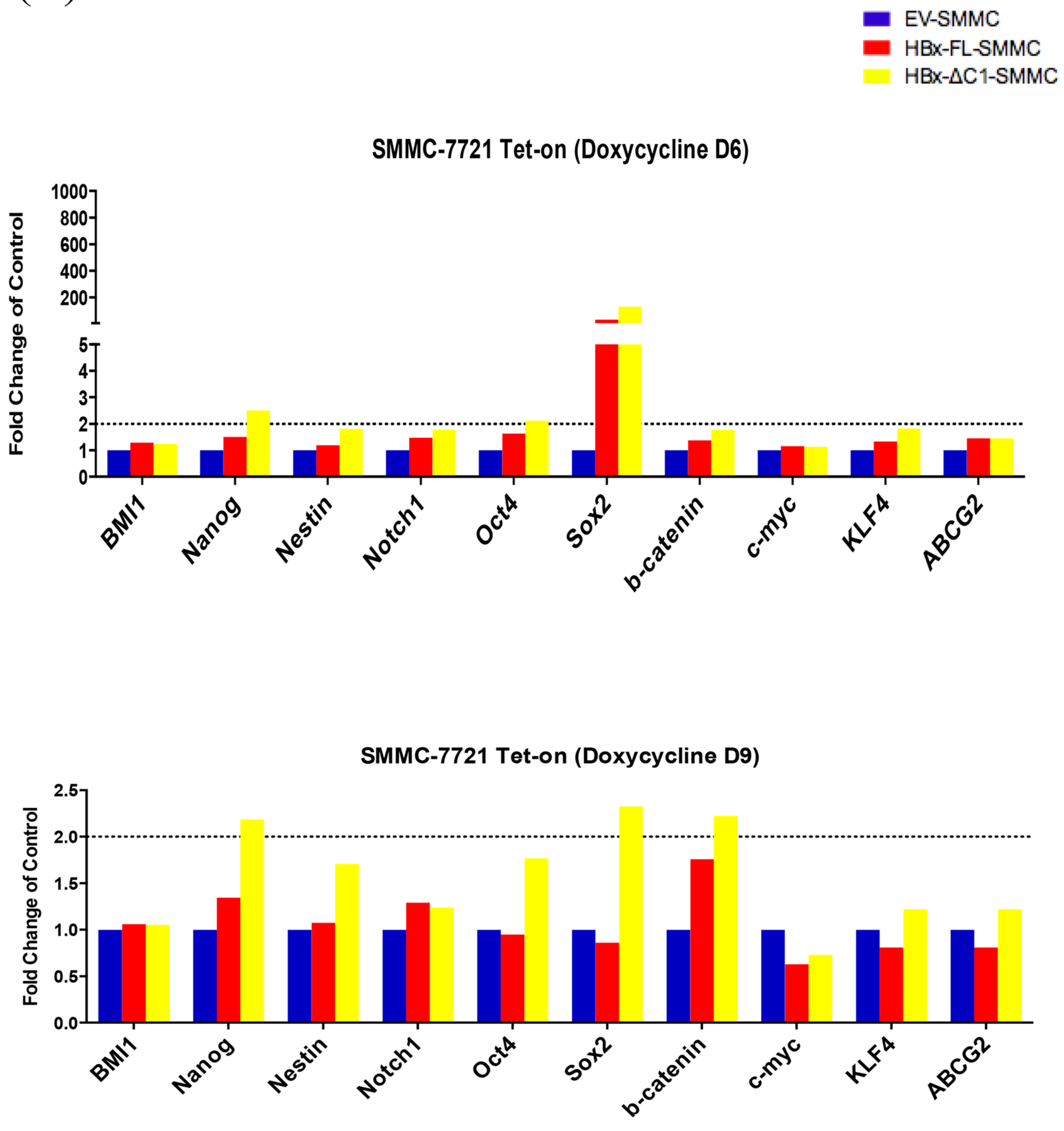

Supplementary Figure 1: Stable inducible expression of HBx-ΔC1 in Bel-7402 and SMMC-7721 enhanced expression of stemness related genes. A. HBx-FL and HBx-ΔC1 were induced by Tet-on Bel-7402 cells, with 1μg/ml doxycycline. By qPCR analysis, it revealed Bel-7402 HBx expressing clones had increased expression of Nanog and Sox2 after adding doxycyclines for 23 days when compared with control (EV). **B.** Unlike Bel-7402, shorter doxycycline incubation time was required to induce the stimulatory effect on expression of stemness related genes in HBx-expressing SMMC cells. After adding doxycycline for 6 days, HBx-FL-Δ and HBx-ΔC1-expressing SMMC-7721 cells had increased mRNA expression of Nanog and Sox2 when compared with EV.

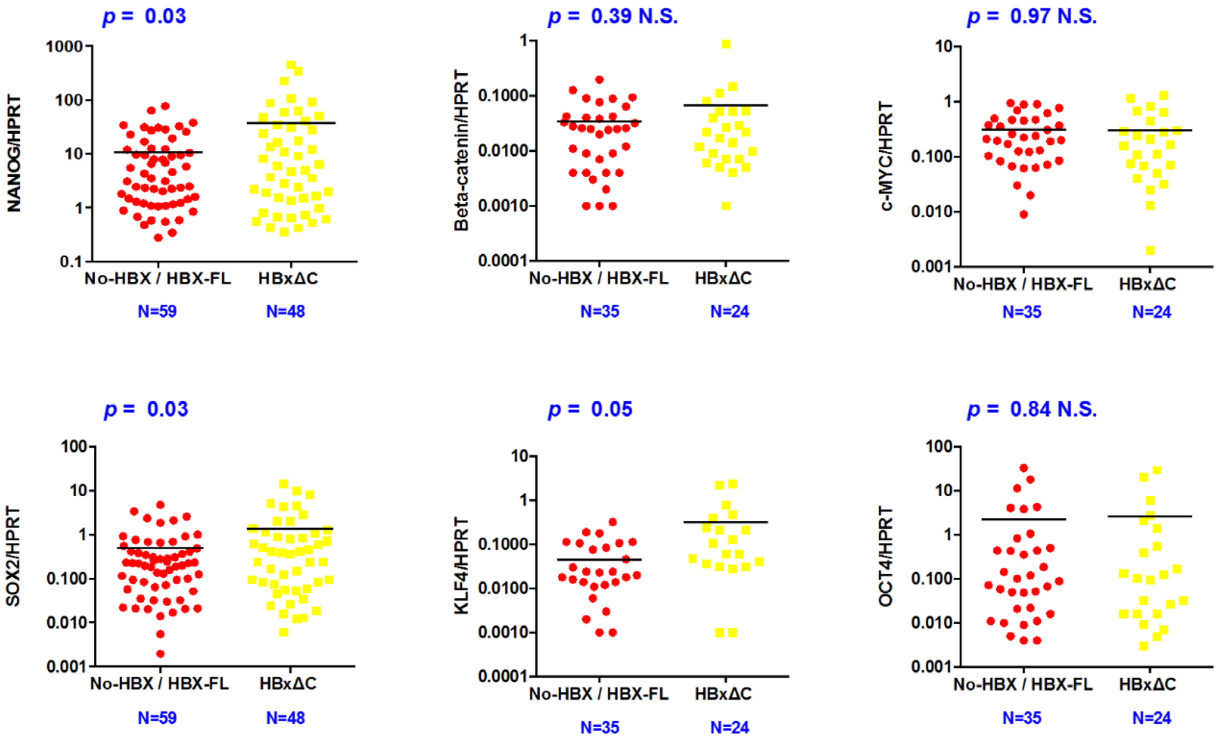

Supplementary Figure 2: Expression of klf-4, Oct4, c-myc, β-catenin, Nanog and Sox2 in non-HBX, HBX-FL and HBX-ΔC related HCC clinical samples. Among these six stemness genes, qPCR analysis demonstrated that the expression of Sox2 and Nanog were up-regulated in HCC samples detected with HBx-ΔC1 (n=48) when compared with those with HBx-negative and HBx-FL (n=59) (**p*=0.03, t test).
